# Reproducibility of Dietary Intake Measurement From Diet Diaries, Photographic Food Records, and a Novel Sensor Method

**DOI:** 10.3389/fnut.2020.00099

**Published:** 2020-07-14

**Authors:** Juan M. Fontana, Zhaoxing Pan, Edward S. Sazonov, Megan A. McCrory, J. Graham Thomas, Kelli S. McGrane, Tyson Marden, Janine A. Higgins

**Affiliations:** ^1^Department of Mechanical Engineering, Faculty of Engineering, National University of Rio Cuarto, Rio Cuarto, Argentina; ^2^National Scientific and Technical Research Council (CONICET), Buenos Aires, Argentina; ^3^Department of Pediatrics, University of Colorado Anschutz Medical Campus, Aurora, CO, United States; ^4^Department of Biostatistics and Informatics, Colorado School of Public Health, University of Colorado Anschutz Medical Campus, Aurora, CO, United States; ^5^Department of Electrical and Computer Engineering, The University of Alabama, Tuscaloosa, AL, United States; ^6^Department of Health Sciences, Boston University, Boston, MA, United States; ^7^Department of Psychiatry and Human Behavior, Alpert Medical School of Brown University, Providence, RI, United States; ^8^Colorado Clinical and Translational Sciences Institute, University of Colorado Anschutz Medical Campus, Aurora, CO, United States; ^9^Department of Pediatrics, Section of Endocrinology, University of Colorado Anschutz Medical Campus, Aurora, CO, United States

**Keywords:** dietary intake, diet diary, food record, photograph, sensor, precision, reproducibility

## Abstract

**Objective:** No data currently exist on the reproducibility of photographic food records compared to diet diaries, two commonly used methods to measure dietary intake. Our aim was to examine the reproducibility of diet diaries, photographic food records, and a novel electronic sensor, consisting of counts of chews and swallows using wearable sensors and video analysis, for estimating energy intake.

**Method:** This was a retrospective analysis of data from a previous study, in which 30 participants (15 female), aged 29 ± 12 y and having a BMI of 27.9 ± 5.5, consumed three identical meals on different days. Four different methods were used to estimate total mass and energy intake on each day: (1) weighed food record; (2) photographic food record; (3) diet diary; and (4) novel mathematical model based on counts of chews and swallows (CCS models) obtained via the use of electronic sensors and video monitoring system. The study staff conducted weighed food records for all meals, took pre- and post-meal photographs, and ensured that diet diaries were completed by participants at the end of each meal. All methods were compared against the weighed food record, which was used as the reference method.

**Results:** Reproducibility was significantly different between the diet diary and photographic food record for total energy intake (*p* = 0.004). The photographic record had greater reproducibility vs. the diet diary for all parameters measured. For total energy intake, the novel sensor method exhibited good reproducibility (repeatability coefficient (RC) of 59.9 (45.9, 70.4), which was better than that for the diet diary [RC = 79.6 (55.5, 103.3)] but not as repeatable as the photographic method [RC = 43.4 (32.1, 53.9)].

**Conclusion:** Photographic food records offer superior precision to the diet diary and, therefore, would be valuable for longitudinal studies with repeated measures of dietary intake. A novel electronic sensor also shows promise for the collection of longitudinal dietary intake data.

## Introduction

Measurement of dietary intake is a necessary but difficult undertaking in clinical and research settings. Common methods used to measure dietary intake include 24-h diet recalls, diet diaries, photographic food records, and food frequency questionnaires (1). There are advantages and disadvantages to each method in terms of cost and participant burden, but all methods share the limitations of self-report. Studies using doubly labeled water have shown that underreporting of food intake is a common problem for self-report methods (2–6). Despite the limitations of these self-report methods, they remain the only validated methods available for measuring dietary intake in free-living situations.

Proper research practice requires that methods be validated against a standard: a previously validated method and/or a biomarker, such as doubly labeled water for energy expenditure (5). Validity refers to the accuracy of any measure; that is, how close the measured value is to the actual value. An equally important, and often overlooked, feature of a method is its reproducibility or precision. Reproducibility or precision is the extent to which a measure yields the same results under similar conditions.

The reproducibility of an instrument is especially important when dietary intake will be recorded longitudinally to assess habitual intake or changes over time. A study using repeated 24-h recalls showed total energy correlation of *r* = 0.59 between measurements (7). Reproducibility research conducted with food frequency questionnaires at two time points has shown that total energy correlations between repeat administrations of questionnaires range from *r* = 0.30–0.92 (7–13). Watson et al. (9) cited under- or over-reporting as a likely contributor to the low reproducibility for the food frequency questionnaire. This concept of systematic under- or over-reporting in dietary assessment was examined by Black and Cole (3). Their review of seven studies with repeated measurements of dietary intake revealed that some persons are more likely to underreport dietary intake than others, regardless of the assessment method used. This personal reporting bias is an issue that should not be ignored when examining dietary intake data and considering the necessity of repeated measures in such research.

Although two previous studies have looked at the reliability of diet diaries, neither used a gold standard reference method, such as a weighed food record, during the same period as the diet diary was recorded, thus limiting the general applicability of the data (14, 15). To our knowledge, no previous study has rigorously examined the reproducibility of the diet diary, which is one of the most commonly used methods to measure free-living dietary intake, or the photographic food record. Both instruments have been studied for accuracy, but there exists no data on their precision. The aim of this study was to examine the reproducibility of diet diaries, photographic food records, and a novel electronic sensor from three separate, identical meals using weighed food records as the gold standard reference method.

## Methods

### Participants

Thirty participants (15 females and 15 males) with a mean (±SD) age of 29 ± 12 y (range: 19–58 y) and body mass index (BMI) of 27.9 ± 5.5 kg/m^2^ (range: 20.5–41.7) were recruited at the Clarkson University campus to participate in the study. The study was approved by the Institutional Review Board at Clarkson University, Potsdam, NY and all participants read and signed an informed consent form before participation. Participants with temporo-mandibular joint disease, dysphagia or other difficulties for chewing and/or swallowing were excluded from the study.

Each participant consumed three full meals at three different visits in the laboratory, ~1–4 weeks apart and at the same clock time at each visit. At the first visit, each participant was asked to select foods according to their own preferences (content and size) from the menu offered by one of the Clarkson University food courts. Any foods or amounts could be chosen by participants, with no restrictions. The initial meal selection was documented so that the selected meal was identical for all three study visits. Participants had no limitations on the quantity of consumed foods or order in which the foods had to be consumed.

### Energy Intake Measurements

Four different methods were used to estimate total mass and energy intake: (1) weighed food record; (2) photographic food record; (3) diet diary; and (4) mathematical models based on counts of chews and swallows (CCS models) obtained via the use of electronic sensors (16).

To obtain the nutritional intake data from meals, records were deidentified and sent to the Colorado Clinical and Translational Sciences Institute's (CCTSI) Nutrition Core. A single operator assessed all deidentified photographic food records and logged consumed food amounts in a standard diet dairy format. A second blinded, independent operator entered all converted photo and original participant food diaries into the nutritional analysis program Nutrient Data System for Research (NDS-R; University of Minnesota, Minneapolis, MN). None of the data entry operators at the CCTSI Nutrition Core were involved in data collection. Using a single, trained operator at each step is the current operating procedure for all CCTSI protocols and reduces variation due to inter-operator differences in data entry. All weighed food records, photographic food records, and diet diaries were de-identified before operator entered nutritional intake into NDS-R. The novel method of using models to count chews and swallows to determine total mass and energy intake was blinded so that operator processing the data was not involved in the post-ingestion annotation of chews and swallow from the original videos.

#### Weighed Food Records

Before and after each meal, food was weighed by a trained member of the research team to calculate the total amount consumed. Each meal was documented and logged into a chart containing detailed information of each food item such as food name and description, mass at beginning and end of the meal, and total mass consumed. For items that could be deconstructed (e.g., a sandwich), each food item was weighed separately before and after consumption. The item was reassembled before being served to the participant. For items that could not be deconstructed (e.g., pizza or cookies) total energy intake was estimated using total weight consumed multiplied by the caloric density of the item. Weighed food records were used as the reference method for actual dietary intake. All other methods were compared to actual dietary intake measured by the weighed food records.

#### Photographic Records

Pre- and post-meal photographs were taken by study staff using a digital camera. The serving plate occupied the entire field of view, and photographs were taken at a 45° angle so that the depth of foods could be estimated (17). A picture of the selected meal was taken before serving and another picture was taken at the end of the eating period. A trained, validated CCTSI nutritionist used these pictures to estimate portion sizes, using the Portion Photos of Popular Foods guide (18) and entered consumed amounts into the food analysis program, NDS-R.

#### Diet Diary

At the beginning of the first visit, participants were trained to complete a diet diary. Oral and written instructions were given to participants for estimating portion sizes and recording foods in sufficient detail to obtain an accurate estimate of dietary intake. Examples of both fully complete and incomplete diaries were explained to demonstrate how to appropriately record intake. Participants also received a portion estimation guide that was used as a reference, but only during the first visit. All materials were supplied by the CCTSI Nutrition Core.

After each meal was finished, participants recorded the food items they just consumed in a blank food diary. Each food item was recorded on a single line indicating the type of food, preparation style, and amount consumed. Participants did not receive any help during this stage; however, the diet diary was reviewed to ensure that it was completed appropriately (i.e., all foods listed had a portion size and description assigned). Participants were not prompted to add any food items they had forgotten to record. Participants only filled out diet diaries for research meals, and no other meals consumed during the 3 days of the study.

#### Models Based on Counts of Chews and Swallows

Estimation of the mass and energy consumed during each meal was computed using participant-dependent models based on counts of chews and swallows. Before starting the experiments, participants were instrumented with a sensor system for monitoring ingestive behavior (19). The system consisted of: (1) a jaw motion sensor placed below the ear to capture chewing events; (2) a miniature microphone placed on the throat to capture swallowing sounds; and (3) a digital camera for video monitoring. Sensor data and video footage were used to compute the number of chews and swallows associated to each meal as previously described (16). The total mass and energy for a given meal was estimated using a counts of chews and swallows model created with the counts of chews and swallows observed in the remaining two meals consumed by the same participant (16).

#### Statistical Analysis

This was a retrospective data analysis of a previous study (16). The sensor method was only analyzed for total energy as this is an exploratory method, still under development and the form described in (16) was only able to estimate mass and energy intake during a meal. When the sensor method is further developed, it will be used to estimate energy, macronutrient, and micronutrient intakes.

Because the actual amount of food consumed varied between study visits, the percent difference from that assessed by the weighed food method serves as the outcome to compare reproducibility across diary, photographic, and sensor methods. The repeatability coefficient (RC) defined as RC $=1.96\times \sqrt{2\;\times }\;SDwithinsubject$ was used to assess the extent of reproducibility for each method. Within-participant variability (SDwithinsubject) of the outcome across three time points was assessed using the with-subject variance from a linear mixed effects model, where the fixed effect consists of intercept only and had a compound symmetry covariance structure. Five thousand Bootstrap samples were based to calculate the 95% confidence intervals for RC for each method and the difference in RC between methods as well as the *p*-values. SAS 9.4 software (SAS Institute Inc.) were used for all the analyses.

## Results

**Figure 1:**
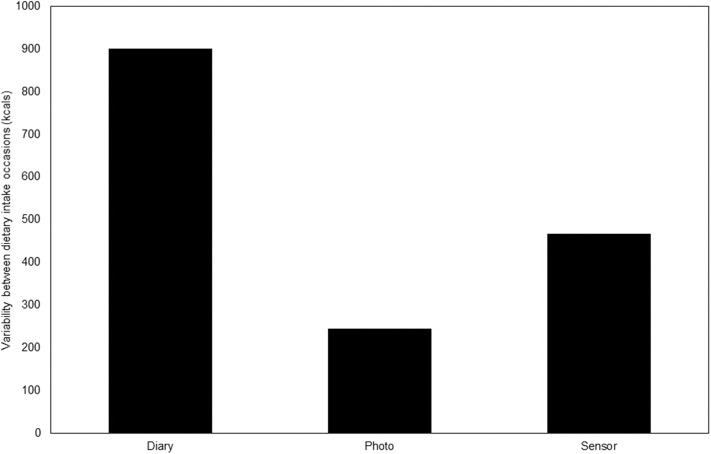
RC and 95% CI of percent difference from weighed method for energy measurements over three time points. The photographic food record and sensor methods had greater reproducibility (lower RC values) than the diet diary for overall energy intake over three time points.

Comparison of the weighed intake data from the three meals indicated that there were no differences in energy or macro- or micro-nutrient intake between the three meals (data not shown). The RC values for the percent difference from the weighed food records revealed that the photographic food record and sensor methods had greater reproducibility [RC = 43.4 (32.1, 53.9) and 59.9 (45.9, 70.4), respectively] than the diet diary [RC = 79.6 (55.5, 103.3)] for total energy intake over three separate meals (Table 1 and Figure 1). Differences in RC values between photographic food records and diet dairies were significantly different for total energy (*p* = 0.004), carbohydrate (*p* = 0.01), protein (*p* = 0.02), calcium (0.004) and iron (*p* = 0.02) intake (Table 2), with photographic food records having greater reproducibility for all nutrients measured (Table 1).

**Table 1:** Repeatability coefficients (95% confidence interval) between measurement methods for percent deviation from weighed measurement.

**Outcome**	**Assessment method**	**RC for percent deviation from weighed measurement^a^**
Total energy (kcal)	Diary	79.6 (55.5, 103.3)
	Photo	43.3 (32.1, 53.9)*
	Sensor	59.9 (45.9, 7.4)
Carbohydrate (g)	Diary	84.1 (56.8, 109.1)
	Photo	42.2 (23.5, 59.0)
Fat (g)	Diary	96.5 (59.4, 136.4)
	Photo	80.6 (48.1, 116.4)
Protein (g)	Diary	99.3 (64.7, 131.2)
	Photo	55.0 (38.3, 70.8)
Fiber (g)	Diary	96.1 (65.9, 123.1)
	Photo	45.2 (28.8, 61.3)*
Calcium (mg)	Diary	93.2 (62.1, 125.2)
	Photo	47.0 (37.8, 55.9)*
Iron (mg)	Diary	188.6 (61.0, 300.2)
	Photo	61.1 (40.8, 79.6)
Sodium (mg)	Diary	224.9 (78.4, 363.3)
	Photo	88.8 (44.2, 134.0)

a *{[Weighed-Diary (or photo)]/weight} × 100*.

* *Statistically significant difference in RCs from diet method at a 5% significant level*.

**Table 2:** Difference in repeatability coefficient between methods over three time points.

**Outcome**	**Comparisons**	**Difference in repeatability coefficient (95%) CL between two methods**	***p*^a^**
Total energy (kcal)	Photo vs. Diary	−36.2 (−63.7, −10.1)	0.004*
	Sensor vs. Diary	−19.6 (−50.7, 8.0)	0.19
	Sensor vs. Photo	16.6 (−2.9, 34.2)	>0.99
Carbohydrate (g)	Photo vs. Diary	−41.9 (−74.7, −8.4)	0.01*
Fat (g)	Photo vs. Diary	−15.9 (−75.0, 39.4)	0.62
Protein (g)	Photo vs. Diary	−44.3 (−83.0, −5.0)	0.02*
Fiber (g)	Photo vs. Diary	−50.9 (−85.6, −15.3)	0.36
Calcium (mg)	Photo vs. Diary	−46.2 (−78.2, −14.3)	0.004*
Iron (mg)	Photo vs. Diary	−127.5 (−230.4, −6.9)	0.02*
Sodium (mg)	Photo vs. Diary	−136.0 (298.3, 6.3)	0.1

a *95% CL and 2-tailed p-values are based on 5,000 bootstrap samples*.

* *P < 0.05*.

## Discussion

Participants completed diet diaries immediately following each meal under supervised conditions. The method of completing the diary immediately post-meal under controlled conditions, as in this study, gives the greatest chance for this method to perform at its best. However, the diet diary method displayed the lowest reproducibility of the three methods tested for total energy intake, and it was inferior to the photographic food record for macronutrients and micronutrients examined. It should be noted that the food photographs were taken by study staff, so this method was also performed under optimal conditions that are not normally present when photographic food records are used.

Reproducibility is an important factor to consider when designing longitudinal studies in which dietary intake is to be measured repeatedly. Under these circumstances, a tool that is more reproducible will decrease the variance in the data collected over time, thereby simplifying data interpretation. Considering the cost and time spent on such studies, as well as participant burden, any instrument which is highly reproducible would add value. Indeed, previous work showed that the photographic food record is as accurate as the diet diary in both energy intake and macronutrient composition but decreases participant burden (17). With the added benefit of higher reproducibility, the photographic food record offers increased utility over the traditional diet diary.

Although two previous studies have looked at the reliability of food records, both compared diet diaries recorded at different times, with no consistency of foods eaten during each recording period (14, 15). Therefore, any differences noted could have reflected actual differences in dietary intake rather than methodological issues. Putz et al. compared two diet diaries to a weighed food record as a reference method (15). However, the weighed food record was completed at separate time from the diet diaries so it is unclear if the dietary intake was similar across occasions and therefore, if the differences measured were due to the method used or actual differences in dietary intake on the different recording occasions. In these previous studies, for estimating total energy intake, the reproducibility of the diet diary was low to moderate [ICC of 0.49 and 0.69 for (14, 15), respectively], which compares well with our estimate of low reproducibility (RC = 43.4). Conversely, we found that the reproducibility for the sensor and photographic record methods was moderate to high, respectively.

Limitations of this study include small sample size, limited age range of participants, that energy intake was not matched between meals for each participant, and photographs in the photographic food records were taken by study staff and not participants. Whereas weighed food records are considered the gold standard and this method was used in our laboratory setting, in a free living situation doubly labeled water could be used to compare reported intake to total energy expenditure, albeit at greater expense. With regard to expense, studies have shown photographic food records to be similar in cost or less costly than self-report methods such as diet diaries and 24-h recalls (20–23). However, when compared to written diet diaries, it does take ~20 more mins per day of intake recording to analyze photos and convert the visual information to amounts for data entry, which is likely irrelevant for smaller studies but could create higher cost overall for large studies. This study had several strengths, however, including the use of a within subject repeated measures design, the large variety of foods for participants to choose from, the use of more than two repeated measures, and that the study took place in a controlled laboratory setting where the researchers had the ability to accurately determine energy intake using weighed food records, considered the gold-standard (24–26).

An interesting finding from this study is that the photographic food record was more precise/reproducible than the sensor method, even though previous work showed that the sensor was more accurate than photographic food records (19). Accuracy may be of greater concern when working with understudied or vulnerable populations where little data currently exist, or in studies that measure dietary intake at a single point. Under these circumstances, the sensor method displays promise, particularly for vulnerable populations such as children with developmental delay or the elderly who may not be able to complete any other method for the estimation of dietary intake. As the sensor can be placed on the participant's jaw and behind the ear, unobtrusively estimating energy intake via measurement of chewing, the need for participant literacy or cognizance of food choices is abolished. In all populations, this method would significantly reduce participant burden and negate some of the pitfalls of self-report. Our future work aims to combine the strengths of the photographic and sensor methods by enabling the sensor to automatically take images of food ingested during the day.

## Conclusion

The higher reproducibility of the photographic food record warrants its use over the diet diary in longitudinal studies which aim to measure dietary intake repeatedly. The novel sensor method for estimating energy intake also shows promise as a dietary intake assessment tool for the future.

## Data Availability Statement

The raw data supporting the conclusions of this article will be made available by the authors, without undue reservation.

## Ethics Statement

This study was approved by the Institutional Review Board at Clarkson University, Potsdam, NY and all participants read and signed an informed consent form before participation.

## Author Contributions

Conceptualization: JF, ZP, ES, MM, and JH. Data curation: JF and TM. Investigation: JF and ES. Project administration: ES, MM, JH, and JT. Supervision: ES, MM, and JH. Writing original draft: TM, JH, and KM. Writing—review and editing: JT, JF, ZP, KM, ES, MM, TM, and JH. All authors contributed to the article and approved the submitted version.

## Conflict of Interest

The authors declare that the research was conducted in the absence of any commercial or financial relationships that could be construed as a potential conflict of interest.

## Supplementary Material

Photographic Records: http://dx.doi.org/10.21227/5mjw-jz09.
